# Innovative strategies for early detection of cardiotoxicity: artificial intelligence and multi-modality collaborative models

**DOI:** 10.1007/s11239-026-03262-y

**Published:** 2026-05-29

**Authors:** Richard C. Becker

**Affiliations:** University of Cincinnati College of Medicine, 231 Albert Sabin Way, Cincinnati, OH 45267 USA

**Keywords:** Cardiotoxicity, Food and Drug Administration, Regulatory bodies

## Abstract

Cancer therapeutics account for a significant proportion of new drug development, reflecting advances in diagnosis, treatment, and disease control. However, with this trend comes a responsibility to ensure patient safety both in the near term and over the long term. Among these considerations, cardiovascular safety is a priority. Downsizing and resource constraints within regulatory agencies, particularly the United States Food and Drug Administration (FDA) threaten safety oversight capacity, increasing the need for automation, structured data standards, common data elements and shared analytics. Public health policy, public–private partnerships, big data capabilities, and the FDA Oncology Center of Excellence (OCE) provide avenues to strengthen Cardio-Oncology safety through cross-center coordination, technology advances, guidance development, and methodological innovation. In this third offering of a 3-part series, the objective is to outline public–private partnerships and collaborative oversight models, linking sponsors, regulators, health systems, payers, and patient groups to operationalize continuous surveillance, standardized cardiac endpoints, and transparent data sharing. Building on existing scientific and regulatory foundations, there is a pressing need to leverage contemporary technologies and creative, interdisciplinary thinking to advance predictive models, monitoring strategies, and mitigation frameworks. A strategic forecasting and preparedness framework is proposed that includes embedding fit-for-purpose cardiac safety endpoints early in development; interoperable, real-time safety data pipelines across pre- and post-marketing phases; deploying artificial intelligence (AI)-enabled signal detection with human-in-the-loop adjudication; using real world evidence (RWE) to confirm clinical benefit-risk under accelerated approvals; and pre-specifying label-update triggers tied to cardiac signal thresholds. Together, these policy and technology innovations can modernize cardiotoxicity detection, protect patients, and sustain benefits in oncology care.

## Introduction

In April 2025, the United States Food and Drug Administration (FDA) underwent a major reduction in force, cutting approximately 3,500 positions with layoffs affecting more than 1 in 6 employees, including senior decision-makers in drug safety and approval are likely to have a substantial negative impact on drug safety assessment, adverse event reporting, and public communication. Fewer staff can lead to delays in drug review, reduced capacity for post-marketing surveillance, and slower dissemination of safety alerts, increasing the risk of undetected adverse drug reactions and eroding public trust in regulatory oversight.

These layoffs affected critical divisions such as the Division of Drug Information (DDI), which lost between 75 and 90% of its staff [1]. This team was responsible for frontline safety monitoring, including handling MedWatch reports, communicating REMS program changes, and responding to safety concerns from healthcare professionals and the public [2]. The cuts may translate to: longer drug review timelines, as fewer reviewers are available to process submissions, delays in safety signal detection, including cardiotoxicity events, due to reduced capacity for post-market surveillance, disruption of REMS programs, which are essential for managing risks of drugs with known toxicities, including cardiovascular risks, and reduced inspection capacity, with the FDA struggling to retain experienced investigators. The vacancies among inspectors rose from 9 to 16% between 2021 and 2024, further limiting oversight of drug manufacturing.

The potential impact is especially concerning for drugs with novel mechanisms or those requiring fast-track review, such as cancer therapeutics with potential cardiotoxic effects. The FDA’s ability to process individual case safety reports (ICSRs) and conduct timely safety assessments has been compromised, raising concerns about delayed identification of adverse drug reactions and limited capacity for timely safety signal evaluation and regulatory action [3].

To address these challenges, the FDA has implemented stricter requirements for post-marketing studies, including more detailed conditions for required studies, frequent sponsor progress reports, and expedited withdrawal proceedings for drugs with unconfirmed benefit, as mandated by recent legislative reforms as summarized in the Code of Federal Regulations (www.ecfr.gov/current/title-21/chapter-I/subchapter-F/part-601/subpart-G/section-601.70; accessed March 13, 2026).

## Opportunities to detect drug cardiotoxicity earlier

### General constructs

Improving the early detection of cardiotoxicity in cancer drug development involves several strategic initiatives. Enhanced preclinical models, including genetically modified animals and advanced in vitro systems such as human-induced pluripotent stem cell-derived cardiomyocytes (hiPSC-CMs), offer more accurate simulations of human cardiac physiology and can predict cardiotoxic effects earlier in the development process [4]. Advanced imaging techniques such as echocardiography, cardiac magnetic resonance imaging (MRI), and Positron Emission Tomography (PET) scans could provide detailed insights into cardiac function and structure during clinical trials, enabling the early identification of cardiac abnormalities and guiding treatment adjustments [5]. The development and expansion of cardiac biomarkers, including high sensitivity troponin and brain natriuretic polypeptide (BNP) are also critical for monitoring cardiotoxicity, with ongoing research focused on discovering more specific and sensitive indicators of cardiac injury [6, 7].

Longitudinal studies that track patients over extended periods are essential for understanding the chronic cardiovascular effects of cancer therapies. Collaborative research initiatives, such as the National Cancer Institute’s Community Oncology Cardiotoxicity Task Force, foster interdisciplinary efforts between cardiologists, oncologists, and researchers to deepen understanding of cardiotoxic mechanisms. Regulatory frameworks must evolve to include more rigorous cardiovascular safety assessments, requiring endpoints in early-phase studies and detailed safety data in new drug applications. Patient-centered approaches that consider individual risk factors can help tailor treatment and standardized monitoring protocols, optimizing the balance between therapeutic efficacy and cardiovascular safety. Education and training for healthcare professionals on cardiotoxicity detection and management, supported by guidelines and best practices, are vital for improving patient outcomes [8].

### Process and policy foundations

Post-approval safety monitoring is critical to ensuring that cancer therapies remain both effective and safe throughout their use. The FDA employs a multi-layered approach, beginning with routine risk-mitigation tools such as safety-related labeling changes, Medication Guides, and postmarketing surveillance requirements; only when these measures are insufficient does the agency implement Risk Evaluation and Mitigation Strategies (REMS) for drugs that pose significant risks, for drugs that pose significant risks, such as certain chemotherapies or radioligand agents. REMS programs may require prescriber certification, restricted distribution, and patient registries to minimize harm and ensure proper use. REMS is a structured program required under the authority of the Food, Drug, and Cosmetic Act (www.fda.gov/about-fda/histories-product-regulation/1938-food-drug-and-cosmetic-act; accessed March 13, 2026), which gives the FDA the legal basis to enforce these risk mitigation measures. The policy represents the statutory and regulatory framework that empowers the FDA to mandate risk evaluation and mitigation, whereas the REMS program is the structured set of tools and actions used to carry out that authority. These measures help balance access to life-saving treatments with the need to prevent severe adverse events.

Under the 21st Century Cures Act (www.fda.gov/regulatory-information/selected-amendments-fdc-act/21st-century-cures-act; accessed March 13, 2026), the FDA established frameworks for continuous review of adverse event reports and scientific literature, particularly during the first three years after approval when unknown risks are most likely to emerge. Periodic audits of REMS compliance ensure accountability, and non-compliance can lead to penalties, label changes, or even withdrawal of the drug from the market. This ongoing vigilance is essential for protecting patients while supporting innovation in oncology.

## What might a new policy to improve safety look like?

A promising policy innovation would be the integration of real-world evidence (RWE) into post-market safety monitoring as a formal requirement. By leveraging electronic health records, claims data, and patient-reported outcomes, regulators could detect emerging safety signals faster and in more diverse populations than traditional clinical trials allow. Coupled with AI-driven analytics, this approach could enable dynamic risk assessment and adaptive REMS programs, ensuring that safety measures evolve alongside new data. Such a policy would not only enhance patient protection but also build public trust in accelerated approval pathways.

Several public health policies are directly aligned with cancer drug development and safety, particularly regarding the prevention, monitoring, and management of cardiotoxicity.

The FDA mandates post-market safety monitoring for all approved cancer drugs, with frequent issuance of black box warnings and safety communications for cardiovascular risks such as heart failure, arrhythmias, and hypertension. Over half of novel cancer therapies have received cardiac safety warnings, reflecting a policy of ongoing risk assessment and communication to clinicians and patients [9].

## Early detection of cardiotoxicity in cancer drug development

### A public–private partnership approach

Early detection of cardiovascular adverse effects is critical to improving patient outcomes and is best achieved in a collaborative framework involving regulators, academia, and industry. Clinical trial investigators, pharmaceutical companies, and regulatory agencies should collaborate by implementing standardized, prospective cardiovascular safety monitoring throughout drug development and post-approval for new cancer therapies [10].

During early-phase and pivotal clinical trials, consensus definitions for cardiovascular endpoints such as heart failure, left ventricular dysfunction, arrhythmias, and major adverse cardiovascular events must be incorporated into trial protocols and reporting systems, with event adjudication by multidisciplinary teams including cardiologists and oncologists [11]. The use of standardized imaging (e.g., echocardiography for LVEF and global longitudinal strain, cardiac MRI for tissue characterization) and serial measurement of cardiac biomarkers (troponin, natriuretic peptides) is recommended by the AHA and ESC to enable early detection of subclinical cardiotoxicity and facilitate cross-trial comparisons [12].

Regulatory agencies should require harmonized cardiovascular safety data collection and reporting and support the establishment of multicenter registries for post-marketing surveillance to capture real-world cardiovascular events, as highlighted in the medical literature [13]. Both the FDA and EMA emphasize the need for harmonized cardiovascular safety assessments. Joint workshops, parallel scientific advice, and shared data platforms are increasingly used to align regulatory expectations. This global alignment facilitates efficient drug development and ensures that cardiotoxicity risks are adequately addressed across jurisdictions (U.S. Food and Drug Administration. (2024). *Scientific public–private partnerships and consortia*. https://www.fda.gov/drugs/science-and-research-drugs/scientific-public-private-partnerships-and-consortia; accessed March 13, 2026).

Pharmaceutical companies should fund and participate in collaborative consortia to develop and validate novel biomarkers, imaging modalities, and machine learning algorithms for risk prediction and early signal detection [14]. Companies must ensure that adverse event reports are complete and timely, as incomplete or delayed submissions can further slow FDA safety assessments.

If under the current executive branch FDA capacity diminishes further, private sector entities will be asked to step in and fill regulatory and safety roles. Amid federal workforce reductions, public–private partnerships (PPPs) offer a pragmatic solution to uphold drug safety and regulatory continuity. The FDA has long engaged in PPPs through initiatives like the *Critical Path Initiative* and collaborations with consortia such as *National Institute for Innovation in Manufacturing Biopharmaceuticals* (NIIMBL) (www.niimbl.org; accessed January 4, 2026) and BioFabUSA a program of the Advanced Regenerative Manufacturing Institute (ARMI) which bring together government, academia, and industry to advance biopharmaceutical innovation and regulatory science (www.armiusa.org/biofabusa/; accessed March 13, 2026). NIIMBL accelerated innovation in biopharmaceutical manufacturing, especially in mRNA and cell therapies, while expanding workforce training nationwide.

BioFabUSA pioneered scalable tissue manufacturing technologies These partnerships have successfully developed tools like predictive biomarkers and streamlined clinical trial methodologies, demonstrating their capacity to fill gaps when federal operations are constrained www.fda.gov/emergency-preparedness-and-response/innovative-technologies/public-private-partnerships; accessed March 13, 2026). There are other examples of successful collaborations that have been forged to meet challenges in drug availability and safety (www.fda.gov/drugs/science-and-research-drugs/scientific-public-private-partnerships-and-consortia; accessed March 13, 2026). New tools, including structured artificial intelligence and machine learning can also be brought to bear for detecting early signals of potential harm [15].

Private equity, when thoughtfully aligned with public health goals, can further strengthen this infrastructure by funding contract development and manufacturing organizations (CDMOs) and clinical research networks within academic research organizations that support drug development and safety oversight. Rather than a unilateral shift of authority, this approach operating in a synergistic environment and a mission-aligned culture emphasizes collaborative governance, transparency, and scientific rigor [16]. PPPs thrive when guided by clear regulatory strategies and shared objectives, ensuring that innovation and safety remain paramount, even in times of federal disruption [17].

Academic societies have a unique opportunity to enhance the dissemination of emerging cardiotoxicity signals by establishing dedicated, frequently updated sections on their websites tailored for oncologists, cardio-oncologists, and other healthcare professionals. These updates could synthesize findings from pharmacovigilance databases, post-marketing surveillance, and real-world evidence studies, offering timely alerts about cardiovascular risks associated with cancer therapies. For example, the American College of Cardiology’s *JACC: CardioOncology* expert panel has published practical recommendations for managing cancer therapy-related cardiovascular toxicity (CTR-CVT), emphasizing the need for surveillance during treatment and individualized risk–benefit assessments for newer immunotherapies such as ICIs [18]. Similarly, the Hellenic Cardiac Society has issued expert guidance on arrhythmias and sudden cardiac death in cancer patients, highlighting the importance of comprehensive cardiologic assessment throughout the cancer care continuum [19]. By centralizing such resources and ensuring regular updates, societies can bridge gaps in clinical practice, support multidisciplinary collaboration, and improve patient outcomes through proactive cardiovascular risk management.

The National Comprehensive Cancer Network (NCCN) and the American Society of Clinical Oncology (ASCO) recommend systematic cardiovascular risk assessment for all cancer survivors, incorporating a review of treatment history, comorbid conditions, and modifiable risk factors. These guidelines emphasize aggressive management of hypertension, dyslipidemia, diabetes, and lifestyle factors, and recommend referral to Cardio-Oncology specialists for individuals at elevated risk. Similarly, the AHA/ACC/HFSA guidelines advise comprehensive pre-treatment screening and surveillance, including baseline assessment of cardiac function before initiating potentially cardiotoxic therapies and continued monitoring during and after treatment to detect early signs of drug-induced cardiomyopathy. Recommended modalities include echocardiography, cardiac biomarkers, and standardized risk calculators, with surveillance intervals tailored to cumulative drug exposure and the patient’s overall risk profile. In addition, contemporary policies support the development of multidisciplinary Cardio-Oncology services designed to coordinate care, streamline monitoring, and improve long-term cardiovascular outcomes in patients undergoing cancer therapy.

The AHA also highlights the need for policies addressing disparities in cardiotoxicity risk and outcomes, including improved representation in clinical trials, population-specific risk assessment, and targeted interventions for high-risk groups such as women and racial/ethnic minorities [20].

## Risk prediction tools from public health and epidemiology databases

Public health and epidemiological databases track cancer incidence, prevalence, and concomitant cardiovascular disease (CVD) rates and are increasingly recognized as essential tools in drug development and safety assessment. These databases provide real-world evidence (RWE) that complements clinical trial data, offering insights into patient populations, comorbidities, and long-term outcomes that are often underrepresented or excluded in controlled studies.

One of the key contributions of these databases is the identification of cardiovascular risks associated with cancer therapies [21]. Epidemiological studies also help quantify the burden of CVD among cancer patients and survivors. A cross-sectional study using data from the National Health and Nutrition Examination Survey (NHANES) found that 21.2% of cancer patients had CVD, with higher prevalence among older adults and males. Independent risk factors included age, smoking, hypertension, diabetes, chronic kidney disease, and antineoplastic treatments [22]. These insights inform drug developers about the need for tailored therapies and safety protocols for populations with dual disease burdens.

Population-based cohort studies further demonstrate the elevated risk of cardiovascular events among cancer survivors. A UK Biobank study found a 51% higher hazard of CVD among cancer survivors compared to non-cancer individuals, even after adjusting for competing risks. A meta-analysis of 104 cohorts confirmed a 1.34-fold increased CVD risk across multiple cancer types and cardiovascular outcomes, independent of chemotherapy or radiotherapy exposure [23]

These findings have significant implications for drug development. They highlight the need for broader trial eligibility criteria, inclusion of cardiovascular endpoints in early-phase studies, and the use of real-world data to validate safety profiles. The FDA has increasingly embraced RWE derived from electronic health records, claims data, and disease registries to support regulatory decisions, including post-market safety monitoring and approval of new indications [24].

Moreover, these observations have directly informed the development of risk prediction tools and screening algorithms. NCCN and ESC/HFA-ICOS risk stratification models incorporate pre-existing CVD and traditional risk factors to categorize patients as low, medium, high, or very high risk for cardiotoxicity [25]. Most validated models use cardiovascular disease history as a key variable; however, recent meta-analyses show that these tools may underestimate risk, especially in patients without overt cardiovascular disease, highlighting the need for improved, cancer-specific prediction models [26].

To optimize information on cardiotoxicity in early phase clinical trials while maintaining a focus on efficacy, trial designs should incorporate: (1) prospective cardiac risk stratification using baseline imaging and biomarkers, (2) serial monitoring for subclinical and clinical cardiac dysfunction throughout the trial, and (3) adaptive statistical models that account for late-onset toxicity and efficacy endpoints, such as Bayesian designs (e.g., TITE-BOIN12- TITE = Time-to-Event; BOIN = Bayesian Optimal Interval;12 = Refers to an extension of the BOIN design that incorporates both toxicity and efficacy outcomes in a two-stage trial) developed by the MD Anderson cancer Center, which allow for real-time decision-making even when toxicity or efficacy outcomes are pending [7].

This integrated approach, spanning preclinical modeling, standardized endpoint definitions, prospective biomarker and imaging surveillance, multidisciplinary adjudication, and post-marketing registries enables the earliest possible identification of cardiotoxicity signals and supports safer, more effective cancer therapy development [27].

### Clinician responsibilities

While efforts to revamp processes, procedures, and policies for earlier detection of cancer drug-induced cardiotoxicity are essential, clinicians must remain vigilant in adhering to established guidelines from national and international societies. Organizations such as the ESC, ASCO and the International Cardio-Oncology Society (IC-OS) recommend a risk-based, multimodal surveillance strategy that includes baseline and follow-up assessments using echocardiography (especially global longitudinal strain), cardiac biomarkers like troponins and natriuretic peptides, and clinical evaluation. These guidelines emphasize the importance of early detection, often during asymptomatic phases and timely intervention with cardioprotective therapies such as ACE inhibitors and beta-blockers to prevent irreversible cardiac damage. As new therapies emerge and survivorship increases, strict adherence to these protocols ensures that innovations in cancer care do not come at the cost of cardiovascular health (Table 1).

**Table 1:** National and international society-based guidelines for cardiotoxicity screening and detection

Cancer therapy class	Society/guideline	Baseline testing	Surveillance during therapy	Post-therapy follow-up
Anthracyclines	ASCO, AHA, ESC, ASE/EACVI	Echocardiogram (LVEF, strain), troponin, BNP/NT-proBNP	Echocardiogram after 250 mg/m^2^, troponin after each cycle, repeat imaging for high risk	Echocardiogram at 6–12 mo, 2 y, and periodically; annual CV assessment
HER2-targeted agents	ASCO, AHA, ESC, ASE/EACVI	Echocardiogram (LVEF, strain), troponin, BNP/NT-proBNP	Echocardiogram every 3 mo, troponin after each cycle	Echocardiogram 6 mo after completion if prior anthracycline; indefinite surveillance for metastatic disease
Immune checkpoint inhibitors	AHA, ESC	Echocardiogram, troponin, BNP/NT-proBNP	Troponin at baseline and before each cycle, imaging if symptoms	Imaging and biomarkers if new cardiac symptoms
VEGF/tyrosine kinase inhibitors	ESC, AHA	Echocardiogram, BNP/NT-proBNP	Periodic echocardiogram, BNP/NT-proBNP during therapy	Imaging and biomarkers if symptoms develop
Proteasome inhibitors	ESC, AHA	Echocardiogram, BNP/NT-proBNP	Periodic echocardiogram, BNP/NT-proBNP during therapy	Imaging and biomarkers if symptoms develop

ASE: American Society of Echocardiography; AHA: American Heart Association; ESC: European Society of Cardiology; EACVI: European Association of Cardiovascular Imaging; ASCO: American Society of Clinical Oncology; LVEF: Left Ventricular Ejection Fraction; BNP: B-type Natriuretic Peptide, NT-proBNP: N-terminal pro B-type Natriuretic Peptide, CV: Cardiovascular

## What is the potential role of artificial intelligence?

Artificial intelligence (AI) will likely play a role in cardiotoxicity detection through real-time monitoring in cancer patients. Machine learning models can analyze vast datasets, including electronic health records, imaging, and wearable device data to predict the likelihood of cardiotoxic events, often identifying subtle patterns missed by traditional methods [28]. Multi-omics integration, combining genomic, proteomic, and metabolomic data, further enhances risk stratification and biomarker discovery, allowing for individualized treatment plans that balance cancer efficacy with cardiovascular safety. AI-enhanced imaging techniques, such as echocardiography and cardiac MRI, may improve diagnostic precision, while wearable and implantable devices enable continuous cardiac surveillance and predictive analytics [29, 30].

While validation methods for AI such as ground truth comparisons, cross-validation and hold-out testing, explainability and interpretability, robustness and stress testing, and bias and fairness audits are available, a detailed discussion is beyond the scope of the series.

## Modernizing cardiac safety: policy, process, and innovation

Emerging technologies may represent the best ways to optimize care in cancer therapeutics. As briefly summarized, AI may be a critical component given its ability to interrogate and structure a vast amount of data from widely disparate sources and use predictive modeling that integrates genomic, proteomic, and imaging data to anticipate cardiotoxicity risks before clinical trials, enabling early intervention and personalized therapy design [31].

In parallel, digital twin technology allows virtual simulations of drug-cardiac interactions, enabling toxicity mitigation strategies without exposing patients to risk [32, 33]. Finally, wearable biosensors enable continuous monitoring of cardiac function during cancer therapy, supporting dynamic risk assessment and early detection of toxicity in real-world settings [34].

**Fig. 1:**
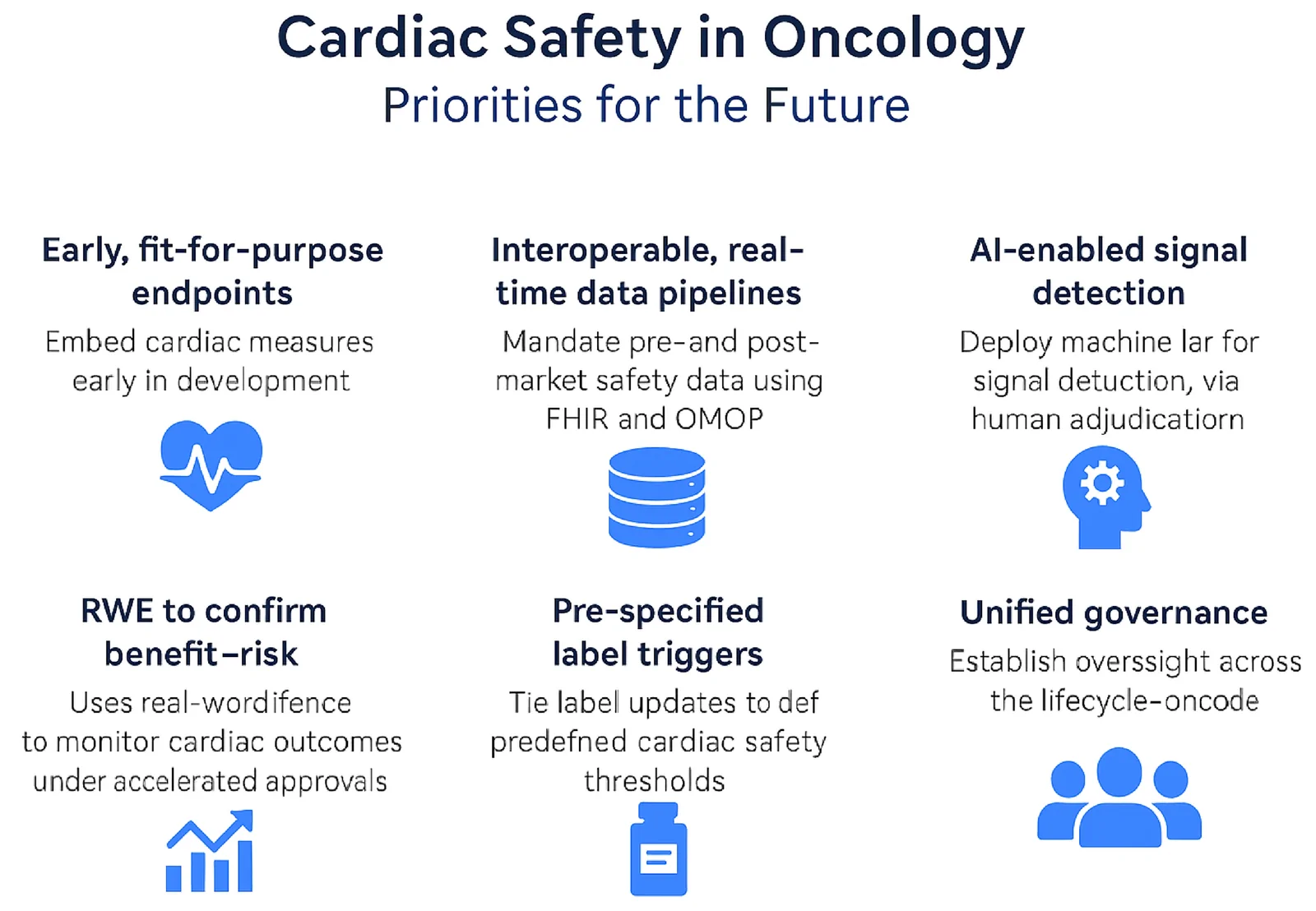
This figure illustrates six strategic priorities for advancing cardiac safety in oncology drug development and lifecycle management. Each box represents a key domain—ranging from embedding fit-for-purpose cardiac endpoints early in trials to establishing unified governance across pre- and post-market phases. The graphic emphasizes integration of interoperable data pipelines, AI-driven signal detection with human oversight, real-world evidence for benefit–risk confirmation, and pre-specified label-update triggers tied to cardiac thresholds. Together, these elements form a forward-looking framework designed to modernize safety processes, protect patients, and sustain innovation in cancer therapeutics

Overall, addressing cardiotoxicity requires a dual strategy: leveraging existing regulatory and scientific infrastructures-such as standardized safety endpoints, pharmacovigilance systems, and real-world data networks while innovating through contemporary technologies and creative policy design (Table 2). By embedding cardiac safety measures early in development, enabling interoperable data pipelines, and integrating AI-driven signal detection with regulatory oversight, one could modernize processes for proactive risk management. Coupled with RWD frameworks and pre-specified label-update triggers, these approaches ensure that oncology innovation is matched by robust safeguards. A theoretical approach and component areas of focus are summarized below (Fig. 1).

**Table 2:** Summary of policy innovations and oversight mechanisms

Initiative	Operational actions	Oversight structure
Endpoint selection	Imaging, biomarkers	Safety monitoring committee
Data pipelines	FHIR/OMOP infrastructure, automated ingestion	Data governance board, audits
AI signal detection	ML models, alert pipelines	Human adjudication team
RWE studies	Propensity-matched RWE, protocol registration	Oversight committee
Label-update triggers	Threshold SOP, regulatory submission	Safety working group

## Early, fit-for-purpose cardiac safety endpoints

Cardiac safety in oncology trials has traditionally relied on core measures such as left ventricular ejection fraction (LVEF), electrocardiographic intervals (e.g., QTc), and periodic imaging (echocardiography or cardiac MRI) to detect declines in systolic function or conduction abnormalities. These methods are well-characterized by Cardio-Oncology consensus guidelines, regulatory body briefs and industry reports. They highlight diverse therapy-specific CV risks, including heart failure, myocarditis, arrhythmias, and hypertension across chemotherapy, targeted agents, and immunotherapies.

Building on this foundation, industry sponsors guided by the FDA, EMA, and clinical research community could embed fit-for-purpose endpoints from first-in-human studies onward, aligning measures to mechanism of action and anticipated CV risk [35]. Protocols would predefine diagnostic timepoints, imaging modality standards, adjudication rules, and stopping criteria. A standing safety monitoring committee, comprising Cardio-Oncology experts, statisticians, and regulatory scientists, would review interim cardiac data at fixed intervals with decision opportunities for dose modification or additional risk minimization. Many of these steps are currently being taken, but formalization and optimization are needed.

## Interoperable, real-time safety data pipelines

Post market pharmacovigilance must continue to evolve from reliance on spontaneous ICSRs toward integration with RWD sources. Modern PV infrastructures increasingly adopt global standards—MedDRA for coding, ICH E2B(R3) for case exchange, and HL7/FHIR and OMOP common data models for data transport and harmonization to improve timeliness and comparability across sources [36].

The process could be optimized by mandating through policies interoperable, continuous data pipelines that connect pre-market clinical databases with post-market EHRs, registries, claims, and device/wearable feeds, using Fast Healthcare Interoperability Resources (FHIR) and Observational Medical Outcomes Partnership (OMOP) for schema alignment and automated ingestion. Governance could include a cross-functional data board overseeing quality controls (deduplication, completeness checks, timestamp verification), quarterly audits, and provenance tracking. This end-to-end architecture reduces signal detection lag, enables longitudinal follow-up, and supports seamless evidence continuity from trials into routine practice [37]. A summary of the FHIR/OMOP system is provided below:

FHIR and OMOP are two widely used standards in healthcare data interoperability and analytics. Initially developed by Health Level Seven (HL7) International, FHIR is a modern standard for exchanging healthcare information electronically. It uses web technologies (REST APIs, JSON, XML) to enable secure, real-time data sharing between systems like electronic health records (EHRs), apps, and registries. FHIR focuses on modular “resources” (e.g., Patient, Observation, Medication) that can be combined for flexible data exchange.

OMOP is a standardized data model designed to harmonize diverse real-world data sources, such as EHRs, claims, and registries into a common structure for large-scale analytics. It supports observational research, safety signal detection, and comparative effectiveness studies by enabling consistent queries across multiple datasets [38].

**Fig. 2:**
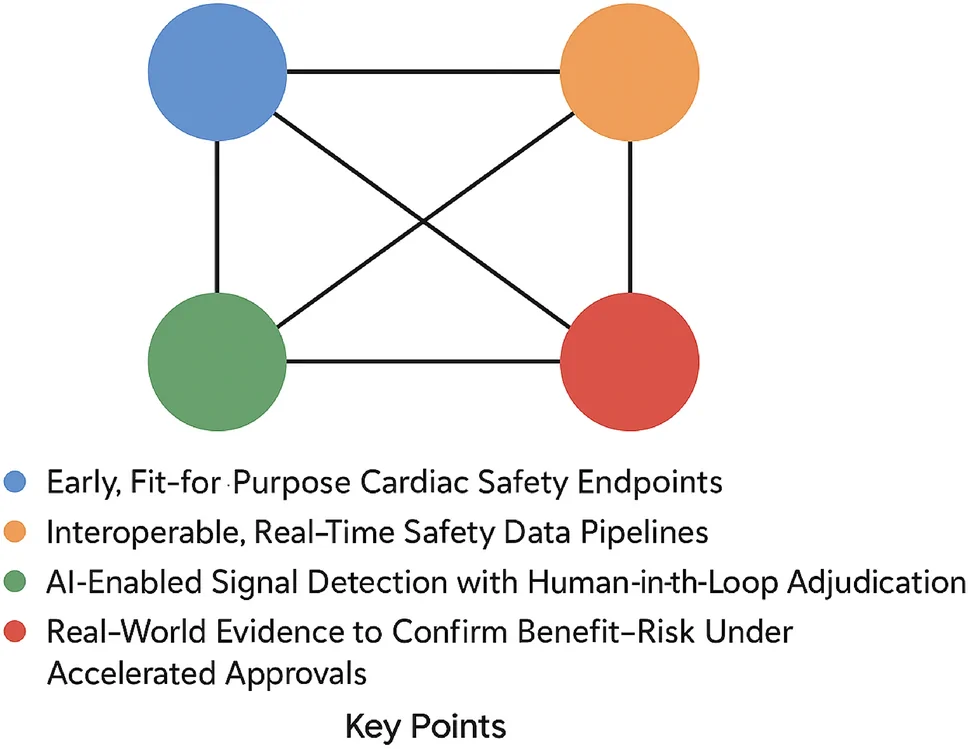
The illustration uses four colored circles to represent key components of modern cardiac safety strategy and their interconnections. **Blue** denotes *Early, Fit-for-Purpose Cardiac Safety Endpoints*, which form the foundation for mechanism-aligned monitoring in trials. **Orange** represents *Interoperable, Real-Time Safety Data Pipelines* (FHIR and OMOP), enabling continuous data flow between premarket and post-market sources. **Green** signifies *AI-Enabled Signal Detection with Human-in-the-Loop Adjudication*, which leverages machine learning and governance for timely, transparent safety insights. **Red** stands for *Real-World Evidence to Confirm Benefit–Risk Under Accelerated Approvals*, ensuring lifecycle validation of oncology therapies. The connecting lines indicate bidirectional relationships: endpoints feed data pipelines, pipelines supply harmonized inputs for AI models, AI informs real-world evidence strategies, and RWE findings loop back to refine endpoints—creating an integrated, iterative system for proactive cardiac risk management

Using FHIR for real-time data exchange and OMOP for harmonized analytics creates an end-to-end pipeline: data flows seamlessly from clinical systems into standardized research environments, enabling interoperability, advanced safety monitoring, and AI-driven insights (Fig. 2).

## AI-enabled signal detection with human-in-the-loop adjudication

AI methods—machine learning (ML) and natural language processing (NLP) are increasingly used to augment signal management across detection, validation, and evaluation steps. Reviews show ML approaches (e.g., random forests, gradient boosting) often outperform traditional disproportionality analyses; NLP improves extraction of clinical context from narratives. Regulatory and multi-stakeholder initiatives are now articulating best practices for transparency, ethics, and common data models [39, 40].

Operationalize AI within PV workflows by training models on structured ICSRs and unstructured clinical text, set calibrated alert thresholds, and routing signals to adjudication teams for clinical causality assessment. Institute periodic external validation against gold-standard datasets, bias and drift monitoring, and auditable model cards for transparency. A “human-in-the-loop” governance structure ensures explainability, ethical safeguards, and regulatory acceptability as AI transitions from experimental pilots to routine PV capability [41].

## Real-world evidence to confirm benefit–risk under accelerated approvals

The 21st Century Cures Act catalyzed regulatory use of RWE, with oncology programs increasingly integrating RWD to evaluate effectiveness and safety across broader, more heterogeneous populations than traditional trials. Methodologies such as external control arms, propensity score matching, and privacy-preserving linkage are now commonly proposed to extend evidence in confirmatory settings and lifecycle management [42].

For accelerated approvals, sponsors should preregister transparent RWE protocols that specify endpoints, data sources, cohort construction, and primary analyses, enabling reproducibility and regulatory review. Real-time dashboards of CV outcomes, including heart failure incidence, myocarditis, and arrhythmias should be monitored by an independent oversight committee with interim decision rules. This approach confirms clinical benefit-risk in diverse populations and can surface rare or delayed cardiotoxicity earlier than passive surveillance alone [43].

## Governance and oversight

Many programs employ safety monitoring committees for trials and PV teams post-approval; yet sustained lifecycle governance linking pre-market and post-market remains heterogeneous and inconsistent. Multi-dataset safety systems combining ICSRs and RWD, supported by AI and data standards could, with further development, emerge as best practice (reviewed in Guha et al. [44]).

**Fig. 3:**
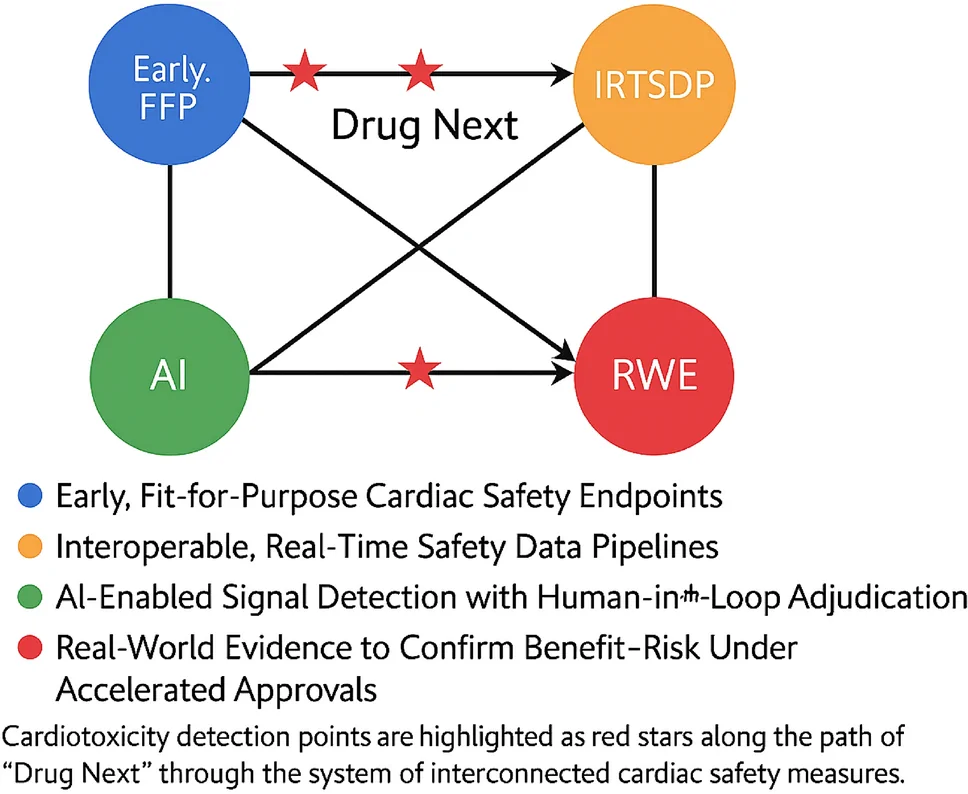
The diagram illustrates the journey of *Drug Next* through the interconnected cardiac safety system. Starting at Early, Fit-for-Purpose (FFP) Cardiac Safety Endpoints (blue), the drug progresses to Interoperable, Real-Time Safety Data Pipelines (IRTSDP) (orange), then to AI-Enabled Signal Detection with Human-in-the-Loop (green), and finally to Real-World Evidence (RWE) for Accelerated Approvals (red). Red stars mark potential cardiotoxicity detection points at each stage-early trial endpoints, continuous data feeds, AI-driven signal alerts, and post-market real-world monitoring, creating a proactive, iterative framework for cardiac risk management

A theoretical example of the process and points of cardiotoxicity detection for a new oncology therapeutic is shown in Fig. 3.

## Aggregate safety assessment planning

Aggregate Safety Assessment Planning (ASAP) offers a flexible foundation for proactive safety oversight. In contemporary settings, ASAP can be integrated with real-world evidence platforms, EHR networks, and AI-driven signal detection tools to enable continuous, cross-study cardiotoxicity monitoring. Linking ASAP with pharmacovigilance databases and decentralized clinical trial infrastructures allows sponsors to harmonize safety data streams, reduce duplication, and accelerate signal validation [45]. Moreover, integrating ASAP with predictive analytics and machine-learning models can further enhance its capabilities, allowing the framework to evolve into a more dynamic and adaptive system that supports real-time risk stratification and early detection of cardiotoxicity.

This integration aligns with regulatory priorities for data transparency, collaborative safety planning, and precision medicine, positioning ASAP as a cornerstone of next-generation safety frameworks [46].

The FDA and European regulators have acknowledged the move toward aggregate safety monitoring in guidance around IND safety reporting, and ASAP’s modular framework aligns directly with this regulatory evolution. Major pharmaceutical companies and CROs have embedded ASAP in their standard safety operations, often incorporating interactive analytics tools such as R Markdown dashboards for near-real-time cross-study reviews [47]. Clinician–scientists are also engaged in ASAP-driven safety teams, ensuring that clinical perspectives help define Safety Topics of Interest (STOIs) and interpret emerging signals.

## Conclusions and future directions

The rapid evolution of cancer therapeutics, particularly targeted agents and immunotherapies, has ushered in a new era of oncologic care, marked by improved survival and disease control. However, this progress has come with a significant and underappreciated cost: cardiotoxicity. As demonstrated throughout this review, cardiovascular adverse events are frequently underestimated during early drug development and only fully recognized years after regulatory approval. This delay poses substantial risks to patient safety, clinical outcomes, and healthcare systems.

Current regulatory frameworks and trial designs inconsistently integrate robust cardiovascular safety measures early in development, and recent reductions in regulatory workforce and capacity further heighten this risk. To address these gaps, cardiovascular safety must become a core element of oncology drug development through early incorporation of fit-for-purpose endpoints, advanced preclinical models, and collaborative oversight involving clinicians, regulators, and industry.

Innovation will play a decisive role in this transformation. AI can enable real-time signal detection and individualized risk prediction, while interoperable data pipelines using FHIR and OMOP standards will ensure seamless integration of trial and real-world evidence. Emerging technologies such as digital twins, multi-omics modeling, and wearable biosensors may offer unprecedented opportunities for proactive monitoring and mitigation of cardiac risk. By embedding these strategies into policy and practice, we can redefine success in cancer care-not only by achieving oncologic efficacy but by safeguarding both near-term and long-term cardiovascular health.

## Summary for the primer

The three-part cardiotoxicity primer emphasized the imperative for cancer therapeutics to achieve a balance between efficacy and safety throughout every stage of development. It laid the groundwork for a system that accelerates progress without compromising thoroughness or sustainability, ensuring that innovation is guided by logic, science, and patient-centered principles.

Central to its framework is the proactive identification of cardiotoxicity risk potential, using advanced tools and predictive methodologies. In the earliest investigation of cardiotoxicity associated with cancer therapeutics, an integrated approach should serve as the foundation for safe development and predictive human phenotyping. This includes elucidating cellular mechanisms of action, leveraging nuclear and tissue biomarkers, applying in silico modeling, and utilizing advanced single-cell or organoid systems. Equally important is the engagement of a broad spectrum of stakeholders-clinicians, researchers, regulators, and industry leaders-working collaboratively to align objectives and share insights.

The primer advocates for integrated processes that leverage regulatory-inspired programs such as Sentinel, which depend on AI, big data analytics, and the strategic use of real-world evidence (RWD). By harnessing data from health systems, registries, and national repositories, these processes enable rapid translation of findings into clinical practice and optimize patient care.

## Data Availability

No datasets were generated or analysed during the current study.
